# Rapid adaptation of the Irish potato famine pathogen *Phytophthora infestans* to changing temperature

**DOI:** 10.1111/eva.12899

**Published:** 2019-12-03

**Authors:** E‐Jiao Wu, Yan‐Ping Wang, Lurwanu Yahuza, Meng‐Han He, Dan‐Li Sun, Yan‐Mei Huang, Yu‐Chan Liu, Li‐Na Yang, Wen Zhu, Jiasui Zhan

**Affiliations:** ^1^ Key Lab for Biopesticide and Chemical Biology Ministry of Education Fujian Agriculture and Forestry University Fuzhou China; ^2^ Fujian Key Laboratory of Plant Virology Institute of Plant Virology Fujian Agriculture and Forestry University Fuzhou China; ^3^ Jiangsu Key Laboratory for Horticultural Crop Genetic Improvement Institute of Pomology Jiangsu Academy of Agricultural Sciences Nanjing China; ^4^ College of Plant Protection Henan Agricultural University Zhengzhou China; ^5^ State Key Laboratory of Ecological Pest Control for Fujian and Taiwan Crops Fujian Agriculture and Forestry University Fuzhou China; ^6^ Department of Forest Mycology and Plant Pathology Swedish University of Agricultural Sciences Uppsala Sweden

**Keywords:** acclimation, aggressiveness, fitness, *Phytophthora infestans*, thermal adaptation

## Abstract

Temperature plays a multidimensional role in host–pathogen interactions. As an important element of climate change, elevated world temperature resulting from global warming presents new challenges to sustainable disease management. Knowledge of pathogen adaptation to global warming is needed to predict future disease epidemiology and formulate mitigating strategies. In this study, 21 *Phytophthora infestans* isolates originating from seven thermal environments were acclimated for 200 days under stepwise increase or decrease of experimental temperatures and evolutionary responses of the isolates to the thermal changes were evaluated. We found temperature acclimation significantly increased the fitness and genetic adaptation of *P. infestans* isolates at both low and high temperatures. Low‐temperature acclimation enforced the countergradient adaptation of the pathogen to its past selection and enhanced the positive association between the pathogen's intrinsic growth rate and aggressiveness. At high temperatures, we found that pathogen growth collapsed near the maximum temperature for growth, suggesting a thermal niche boundary may exist in the evolutionary adaptation of *P. infestans*. These results indicate that pathogens can quickly adapt to temperature shifts in global warming. If this is associated with environmental conditions favoring pathogen spread, it will threaten future food security and human health and require the establishment of mitigating actions.

## 1 INTRODUCTION

The epidemiological development of infectious diseases results from the interaction of three main factors: a conducible environment, a susceptible host, and a virulent pathogen (Madden, Hughes, & Bosch, 2007). Climate change is expected to exert a strong impact on the epidemics and ecology of plant and animal diseases (Garrett, Dendy, Frank, Rouse, & Travers, 2006; Sparks, Forbes, Hijmans, & Garrett, 2014; Zhan, Ericson, & Burdon, 2018), greatly threatening food security, natural landscapes, and human health (Boland, Melzer, Hopkin, Higgins, & Nassuth, 2004; Kalinda, Chimbari, & Mukaratirwa, 2017; Myers et al., 2017). These epidemic effects on infectious diseases can result from the influences of climate changes on the survival, reproduction, and transmission of both pathogens and hosts and can occur at various spatial and temporal scales (Koelle, Pascual, & Yunus, 2005).

Global warming is an important element of climate change of great concern to society. In recent times, the Earth's climate has warmed at an unprecedented rate—a phenomenon set to continue, rising by 4°C above the pre‐industrial era by the end of the 21st century (IPCC, 2014). As a major abiotic environmental factor, temperature can impact on a very broad range of biological and biochemical activities of living organisms from the subcellular molecular level to community‐wide interactions (Huey & Kingsolver, ; Padfield, Buckling, Warfield, Lowe, & Yvon‐Durocher, 2018; Schaum et al., 2017; Yvon‐Durocher et al., 2015). In host–pathogen interactions, temperature can exert a strong influence on the ecology and severity of diseases (Elad & Pertot, 2014), and the evolutionary dynamics of hosts and pathogens (Gillman, Keeling, Gardner, & Wright, 2010). It does this by affecting key stages of the life cycles of either or both hosts and pathogens (Addison, Powell, Six, Moore, & Bentz, 2013; Dysthe, Bracewell, & Six, 2015; Zhan & McDonald, 2011) including survivals (Mariette et al., 2016), reproductive modes (Angilletta, Oufiero, & Leaché, 2006), dispersal abilities (Urban Mark, Tewksbury Josh, & Sheldon Kimberly, 2012), and geographic distribution (Rutherford, D'Hondt, & Prell, 1999) as well as their interactions with other biotic and abiotic elements in the environment (Eastburn, McElrone, & Bilgin, 2011). Given the multidimensional effects of temperature on host–pathogen interactions, a warmer world resulting from climate change may well present new disease management challenges. In this context, it is crucial to analyze the patterns of thermal adaptation of pathogens to future temperature change in order to develop better predictions of future disease epidemiology and formulate relevant management strategies (Mboup et al., 2012).

It is well known that living organisms can adapt to environmental changes by acclimation (Ghalambor, Mckay, Carroll, & Reznickd, 2007). Through prior exposure to a stressing environment for a short period of time, living organisms often show a marked increase in their fitness compared to individuals naive to the stress (Hoffmann, 1995). This adaptation can be driven genetically or physiologically and has been documented widely in pathogens, plants, and animals in the context of temperature stresses (Hoffmann & Willi, 2008). Physiological adaptation to thermal changes caused by phenotypic plasticity does not modify the genetic architecture of living organisms. Rather, it is the near‐instant response of living organisms to environmental stresses achieved through temporal adjustment of gene expression and/or enzymatic activity (de Nadal, Ammerer, & Posas, 2011; Wu, Wang, Shen, et al., 2019). On the other hand, genetic adaptation involves permanent or quasi‐permanent (epigenetic control) changes in the genomic composition of living organisms followed by directional selection favoring beneficial change and usually occurs in addition to physiological adaptation (Hoekstra & Coyne, 2007). The two adaptation processes are interconnected to provide an opportunity for the organism in question to survive, reproduce, and compete in new environments (Willmott et al., 2018). Many empirical studies of thermal adaptation have focused on the scales and patterns of physiological adaptation, that is, phenotypic plasticity (Bacigalupe et al., 2018; Chown, Addo‐Bediako, & Gaston, 2002; Garrett et al., 2006; Willmott et al., 2018). In contrast, evolutionary inferences on how fast genetic adaptation can occur and how such adaptations may affect biological interactions among traits are relatively limited, particularly in plant pathogens. For example, rapidly physiological adaptation to temperature stresses during thermal acclimation has been documented in some animals, plants, and microbes (Colinet, Overgaard, Com, & Sorensen, 2013; Crowther & Bradford, 2013; Donelson, Munday, Mccormick, & Pitcher, 2012; Fahey, Winter, Slot, & Kitajima, 2016), but no such phenomenon has been reported in a pathogen (Paull, Raffel, LaFonte, & Johnson, 2015). All of these reports on thermal adaptation during acclimation were conducted by continuing passage of the concerned species in a fixed temperature regime.

The potato *Phytophthora infestans* association is a particularly relevant one in which to assess the potential for thermal adaptation in pathogens. Potato has been and is playing an ever‐increasing role, as a major food crop (currently ranked as the third largest food crop globally) feeding the world's growing population (Birch et al., 2012). *Phytophthora infestans* is the world's most devastating potato pathogen (Fry, 2008), causing annual economic losses of approximately 8 billion US dollars (Birch et al., 2012; Haverkort et al., 2008; Runno‐Paurson et al., 2013). It infects all parts of the potato plant including leaves, stems, and tubers, and under moderate temperature (16–22°C) and high humidity (over 97%), can destroy entire crops within a few days of infection (Harrison, 1992; Latijnhouwers, Ligterink, Vleeshouwers, Van West, & Govers, 2004; Sujkowski, 1987). While resistant genes are available in cultivated and wild potatoes, most of the resistance is difficult to integrate into modern cultivars and can be quickly rendered ineffective due to evolution in the pathogen (Forbes, 2012; Yang et al., 2019). Consequently, the disease is mainly managed by fungicide applications, causing big challenges to environmental sustainability and biodiversity conservation.

*Phytophthora infestans* is considered to be a heterothallic oomycete with two mating types designated A1 and A2, despite the existence of self‐fertile isolates (Judelson, 1997). It can reproduce sexually, asexually, and/or parasexually (Billiard et al., 2011; Zhu et al., 2016) and has a potential for long distance wind dispersal (Granke, Windstam, Hoch, Smart, & Hausbeck, 2009). The pathogen has a large genome (~240 Mbp) rich in transposable elements (>75% of total genome) (Haas et al., 2009; Vetukuri et al., 2012). Many genes critical to the ecological functioning of the pathogen are located around these highly mutable regions, providing a unique opportunity to generate large amount of variation available for quick evolutionary adaptation to changing biotic environments such as host resistance (Lozoya‐Saldaña, 2011; Rietman et al., 2012) or abiotic environments such as temperature changes (Mariette et al., 2016). Previous analysis of the association between historical thermal selection and evolution in *P. infestans* found a countergradient adaptation in metabolic rate and fungicide resistance, a trade‐off between temperature niche breadth and intrinsic growth rate, and a greater physiological than genetic contribution to the observed thermal adaptation (Qin et al., 2016; Yang et al., 2016).

Here we move beyond this essentially observational approach by using an experimental evolution (acclimation) approach to understand the patterns of thermal adaptation in *P. infestans*. According to our best knowledge, no such information is available in this important pathogen. Unlike other studies that acclimated a pathogen, plant, or animal to a fixed temperature (Crowther & Bradford, 2013; Wos & Willi, 2018; Zhang et al., 2015), we mimicked gradual increases or decreases in air temperatures in nature by small incremental or decremental “stepping‐stone” changes in experimental temperatures, by 1°C each time, starting from the lower (12°C) and upper (26°C) thermal boundaries usually required for the epidemic development of *P. infestans* in the field (Yang et al., 2016) and are unaware this incremental (decremental) approach has been used to study the thermal acclimation of species in literatures. The isolates, collected from different field sites, representing various evolutionary histories of thermal adaptation of the pathogen were used as parental populations and acclimated continuously for 40 days at each experimental temperature step to increase the chance of mutation and selection.

The specific objectives of the study are as follows: (a) to investigate the pattern of thermal adaptation in *P. infestans* by analyzing its phenotypic responses to changing temperature; (b) to infer the possible mechanisms contributing to the thermal adaptation of *P. infestans* during acclimation; and (c) to analyze the effect of thermal adaptation on the interaction among biological traits of the pathogen.

## 2 MATERIALS AND METHODS

### 2.1 Parental isolates for the temperature acclimation experiment

**Figure 1:**
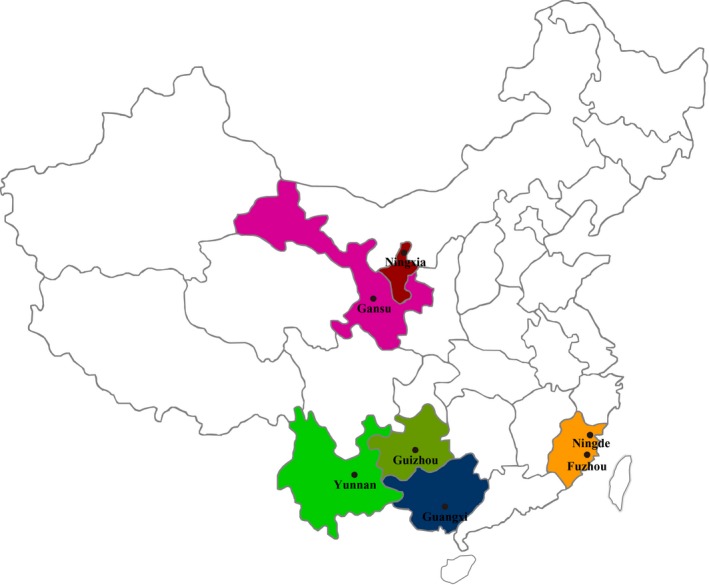
Map showing the geographic locations of the seven *Phytophthora infestans* populations included in this study

A total of 21 genetically distinct *P. infestans* isolates previously characterized by SSR assays (Lees et al., 2006; Qin et al., 2016; Wu, Wang, Shen, et al., 2019) and sequence analysis of several functional genes (Cárdenas et al., 2011; Yang et al., 2018) were selected for the temperature acclimation experiment. These isolates were randomly collected from seven potato fields (three isolates from each field) located in Gansu, Guangxi, Guizhou, Ningxia, Yunnan, and Fujian (Fuzhou and Xiapu) along a climatic gradient of China at the early stages of epidemics in 2010 and 2011 (Figure 1, Wu, Wang, Shen, et al., 2019). Among these locations, Gansu and Ningxia, representing a continental climate, and Guizhou and Yunnan, representing a temperate climate, are the four top potato production areas in China, while Guangxi and Fujian, representing a subtropical climate, are the two provinces with the highest potential of developing a potato industry in coming decades (Yang et al., 2016). Detailed information on the collection, isolation, and molecular characterization of the pathogen isolates is provided in our previous publications (Qin et al., 2016; Wu, Wang, Shen, et al., 2019).

### 2.2 Temperature acclimation

**Figure 2:**
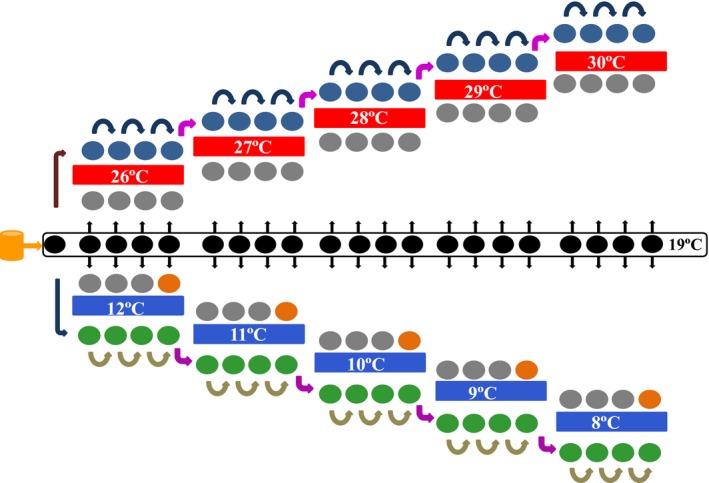
The flowchart of *Phytophthora infestans* temperature acclimatization: The yellow cylinder represents the parental isolates preserved in microtubes at 13°C for long‐term storage. The black circles represent the parental isolates revived and maintained on rye B plates at 19°C (the optimum temperature of the pathogen); gray circles represent parental isolates without trained (controls); blue circles represent isolates continuously trained under increasing temperatures; and green circles represent isolates continuously trained under reducing temperatures. Arrows represent the transfer of isolates to fresh rye B plates after 10 days (one generation), and orange circles indicate the stages when aggressiveness of acclimated isolates was tested. The acclimation lasted for 20 generations (transfers) with 10 days in each generation

*Phytophthora infestans* isolates revived from long‐term storage were acclimated under a pattern of stepwise increases or decreases of temperature over a total of 20 generations (10 days per generation) for 200 days on rye B agar (50 g/L rye and 12 g/L agar) in 9‐cm petri dishes (Figure 2). In the high‐temperature acclimation experiment, pathogen isolates were grown under a 1°C incremental sequence of increasing experimental temperatures starting at 26°C and ending at 30°C (five temperature regimes in total). At each temperature step, pathogen isolates were acclimated for four generations (40 days = 10 days/generation). At the end of each generation, the colony size of each isolate was digitalized and a mycelial plug (*ϕ* = 5 mm) taken to initiate a new generation on a fresh rye B agar plate. Similarly, in the low‐temperature acclimation, pathogen isolates were subject to a steady 1°C stepwise decrease of experimental temperatures starting at 12°C and ending at 8°C (again a total of five temperature regimes). The low and high thermal limits were chosen to reflect extreme temperature regimes to which, our previous studies have shown one or more of the pathogen populations was exposed during the potato growing season (i.e., during the pathogenic phase of its life cycle) across the sampled climatic gradient. Globally, most potato‐growing regions fall within these extreme cold and hot limits of temperatures (Haverkort, 1990). For both the low‐ and high‐temperature acclimations, controls were set by directly transferring the unacclimated parental isolates maintained in the long term at the pathogen's optimum of 19°C (Yang et al., 2016) to the current acclimation temperature regime anew at the beginning of each generation (Figure 2, gray circles). Control plates inoculated with unacclimated parental isolates were cultured together with the acclimated isolates in each temperature regime. The experimental units (isolates) of both acclimated isolates and unacclimated controls at each temperature regime were repeated three times (i.e., grown on three petri dishes filled with rye B agar), batched together according to temperature regimes and laid out on a completely randomized design in the same growth chamber supplemented with lights. To minimize experimental errors, the entire inoculation procedure associated with the particular temperature regime was completed by the same person in the same day.

### 2.3 Colony growth and aggressiveness measurement of acclimated and unacclimated parental *Phytophthora infestans* isolates

Colonies of *P. infestans* isolates were photographed using a Nikon‐26 camera at the end of each generation (10th day after inoculation) for both acclimated and unacclimated parental isolates. Plates with concerned *P. infestans* colonies were placed 60 cm under the camera mounted on a tripod, creating images with a resolution of ~300 dpi. Artificial lights produced by a fluorescent lamp were used for photographing. As a result, a total of 5,040 [21 isolates × 3 repeats × 4 generations × 10 temperatures (5 low temperatures + 5 high temperatures) × 2 treatments (1 acclimation + 1 unacclimated)] colonies were digitalized in the experiment and their sizes were estimated using the image analysis software ASSESS (Lamari, 2002). The software measures colony area (cm^2^) based on the number of pixels inside the delimited polygon. For most of images, the software can recognize the boundaries between agar plates and pathogen colonies automatically and determine the delimited polygon for the colonies using the color planes embedded. Some manual manipulations might be required for the images with low contrast such as those taken from thinner colonies. Aggressiveness of the isolates was evaluated at the end of each four generations at a particular temperature regime by measuring lesion development on detached potato leaves (Zhan et al., 2005). Leaf‐based aggressiveness tests were performed on detached leaflets taken from the potato cultivar Favorita, a cultivar universally susceptible to *P. infestans* (Foolad, Sullenberger, & Ashrafi, 2015). To do this, fully expanded leaflets excised from Favorita plants grown in a disease‐free field for about 8 weeks were placed in petri dishes (*ϕ* = 9 cm, where *ϕ* represents diameter) filled with 2% water agar and then inoculated on the abaxial side with mycelial plugs (*ϕ* = 5 mm) taken from the colonies of both the acclimated and unacclimated parental isolates at the end of the last generation (i.e., 10th day of the 4th, 8th, 12th, 16th, and 20th generations) of each temperature regime. The petri dishes containing the inoculated leaflets were kept in a growth chamber with a programmed temperature corresponding to the temperature regimes of the acclimation experiment. For example, if the assay was tested for the aggressiveness of isolates taken from the last generation of acclimation at 10°C, the temperature in the growth chamber was also programmed to 10°C. Disease lesions formed on inoculated leaflets were photographed 10 days after inoculation. Lesion sizes were also measured by the image analysis software ASSESS (Lamari, 2002) and used as an indicator of pathogen aggressiveness. Aggressiveness measured by growth on potato leaflets was tested for all acclimated and unacclimated parental isolates from both high‐ and low‐temperature acclimation experiments. However, only data from the low‐temperature acclimation experiment were available for further analysis of thermal adaptation as the high‐temperature acclimation experiment failed because most leaflets held under the corresponding temperature regime rotted before disease lesions appeared. As a result, a total of 840 disease data points were generated [21 isolates × 4 replicates × 5 experimental temperatures × 2 treatments (1 acclimation + 1 unacclimated)] and included in the further analysis of thermal adaptation in the pathogen. As for colony growth assessments, the entire measurements of *P. infestans* aggressiveness were completed by the same person to reduce any possible errors associated with selection of plant materials, inoculation procedure, image analysis, etc.

### 2.4 Data analysis

Fitness of the *P. infestans* isolates was inferred from the amount of disease (aggressiveness) they induced on the detached leaflets of the susceptible potato cultivar Favorita and/or colony sizes they formed on rye B agar after 10 days of inoculation. Thermal adaptation of the pathogen was evaluated by comparing the fitness of the acclimated isolates relative to their unacclimated parental isolates in each generation and temperature regime as well as the average across generations and temperature regimes. Analysis of variance (ANOVA) for colony size and aggressiveness was conducted using a general linear model (SAS Institute, 2013) according to the following:

$$Y_{\text{matpi}}=M+A+T+P+\text{I(P)}+E_{\text{matpi}}$$

where Y_matpi_ is the mean colony size or aggressiveness for isolate i(p) from population p at temperature under treatment A and M, A, T, P, I(P), and E_matpi_ are the overall mean, treatment (acclimated isolates vs. unacclimated parents), experimental temperature, pathogen collection sites, isolate, and the variance among replicates, respectively. In this analysis, variance was estimated separately for high‐ and low‐temperature acclimations and temperature and collection site were treated as fixed variable while isolates and treatment were treated as random variables. Duncan's multiple range and LSD tests (Ott, 1992) were applied to the results to determine the significance of differences in fitness between the acclimated and unacclimated parental isolates and among isolates originating from locations differing in annual thermal conditions based on the SAS software. Monthly temperature data presented as an average over 15–30 years for each collection site were downloaded from World Climate (http://www.world-climate.com/) as described previously (He et al., 2018; Yang et al., 2016; Zhan & McDonald, 2011). Association between colony size and aggressiveness in both the acclimated and unacclimated parental isolates was evaluated by Pearson's correlation (Lawrence & Lin, 1989). In this analysis, colony size data of the isolates from different generations within the same temperature regime were pooled together. Pearson's correlation was also used to evaluate the association between fitness components (colony size and aggressiveness) and thermal condition at the pathogen collection sites as well as the impact of temperature acclimation on this association. Colony size and aggressiveness averaged over different generations of the same temperature regimes as well as different isolates derived from the same location were used for this analysis.

## 3 RESULTS

Temperature acclimation significantly increased *P. infestans* fitness, measured by aggressiveness on detached leaves and/or colony size on agar (*p* < .05), although isolates from different geographic locations varied in the extent of the improvement (Tables 1 and 2). Under high‐temperature acclimation, the mean colony size across the 20 generations in the seven populations ranged from 7.16 to 16.06 cm^2^ with an overall average of 11.16 cm^2^ in the acclimated isolates compared to an overall average of 9.30 cm^2^ with a range of 4.42 to 16.38 cm^2^ in the unacclimated parental isolates. Under low‐temperature acclimation, the mean colony size and aggressiveness (lesion size) in the seven populations ranged from 19.33 to 30.74 cm^2^ and 2.24 to 8.71 cm^2^ with an overall average of 25.76 and 5.86 cm^2^ in the acclimated isolates compared to an overall average of 21.36 and 4.58 cm^2^ with a range of 15.54–27.48 cm^2^ and 1.07–7.43 cm^2^ in the unacclimated parental isolates, respectively. In most cases of high‐ and low‐temperature acclimations, trained isolates performed better than untrained parental isolates (Tables 1 and 2).

**Table 1:** Duncan's multiple range tests for differences in colony size and aggressiveness among acclimated and unacclimated *Phytophthora infestans* isolates originated from different temperature zones of China

Groups	AMT (°C)	Colony size at low‐temperature acclimation (cm^2^)		Colony size at high‐temperature acclimation (cm^2^)		Lesion size at low‐temperature acclimation (cm^2^)	
		Unacclimated	Acclimated	Unacclimated	Acclimated	Unacclimated	Acclimated
Guizhou	14.7	21.83 ± 1.47^B^	29.01 ± 0.94^B^	9.35 ± 1.72^C^	10.72 ± 1.75^C^	6.74 ± 1.44^A^	7.17 ± 1.13^A^
Fuzhou	20.5	27.48 ± 2.48^A^	30.74 ± 1.64^A^	16.38 ± 2.45^A^	16.06 ± 2.36^A^	3.77 ± 1.11^CD^	5.43 ± 1.36^B^
Guangxi	22.6	17.38 ± 1.43^C^	19.33 ± 1.15^F^	11.91 ± 2.07^B^	13.55 ± 1.84^B^	1.07 ± 0.98^E^	2.24 ± 0.98^C^
Gansu	11.7	23.09 ± 1.59^B^	30.29 ± 0.84^AB^	8.39 ± 1.69^CD^	11.41 ± 1.59^BC^	7.43 ± 1.39^A^	8.71 ± 1.15^A^
Ningxia	7.0	22.82 ± 1.48^B^	25.91 ± 1.12^C^	8.30 ± 1.49^CD^	11.12 ± 1.52^C^	4.73 ± 1.36^BC^	5.32 ± 1.27^B^
Xiapu	20.3	15.54 ± 1.19^C^	21.38 ± 0.80^E^	4.42 ± 0.79^E^	7.16 ± 0.93^D^	2.64 ± 1.23^D^	4.52 ± 1.22^B^
Yunnan	15.6	21.37 ± 1.38^B^	23.67 ± 0.87^D^	6.36 ± 0.95^DE^	8.12 ± 1.05^D^	6.12 ± 1.29^AB^	7.64 ± 1.27^A^

Note

Values followed by different letters in the same column are significantly different at *p* = .05, and data in parentheses are 95% confidence interval.

Abbreviation: AMT, average mean temperature.

**Table 2:** Comparison of colony size and aggressiveness between acclimated and unacclimated *Phytophthora infestans* isolates

Treatments	Colony size at low‐temperature acclimation (cm^2^)	Colony size at high‐temperature acclimation (cm^2^)	Lesion size at low‐temperature acclimation (cm^2^)
Acclimated	25.76 ± 0.47^A^	11.16 ± 0.63^A^	5.86 ± 0.48^A^
Unacclimated	21.36 ± 0.46^B^	9.30 ± 0.66^B^	4.58 ± 0.49^B^
Gain (%)	20.60	20.00	27.95

Note

Values followed by different letters in the same column are significantly different at *p* = .05, and data in parentheses are 95% confidence interval.

**Figure 3:**
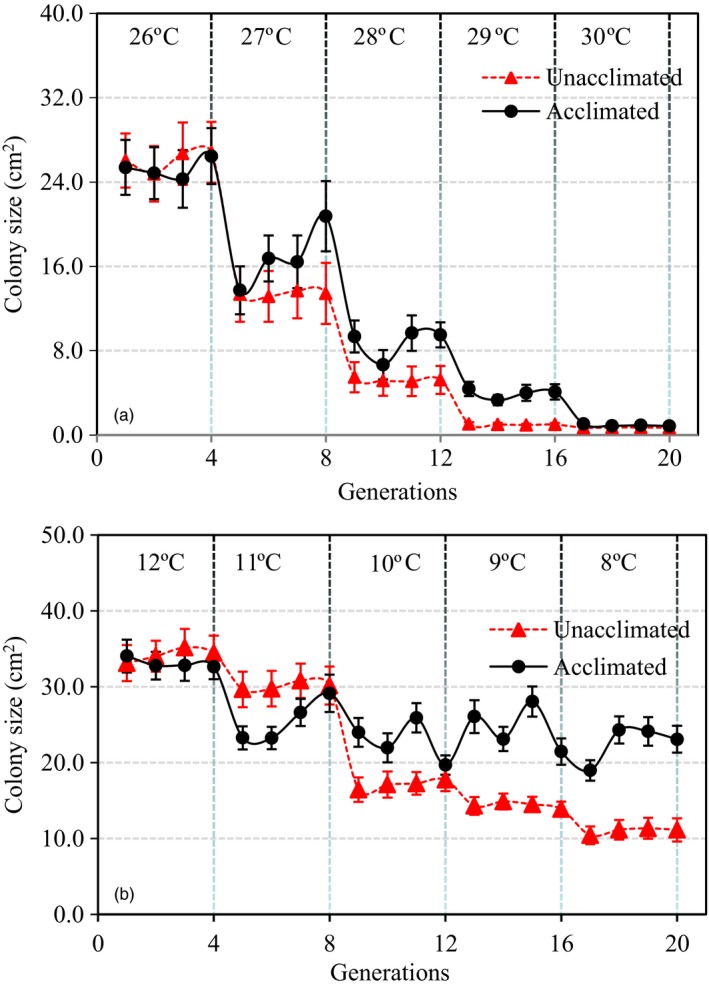
Temporal changes (fitted smoother) of mean colony size and its 95% confidence interval (CI) in the acclimated and unacclimated *Phytophthora infestans* isolates. The mean colony sizes were estimated over all 21 isolates collected from the seven locations. Acclimation was carried out in two directions under continuing increase or decrease of temperatures over 20 generations (10 days per generation). At the high‐temperature acclimation, the pathogen was continually trained under a steady increase of experimental temperatures at 1°C interval starting from 26°C and ending at 30°C (total five temperature regimes). In each temperature regime, the pathogen was acclimated for four generations. Similarly, at the low‐temperature acclimation, the pathogen was continually trained under a steady decrease of experimental temperatures at 1°C interval starting from 12°C. The acclimation was also executed under five temperature regimes (ending at 8°C) each with four generations: (a) high‐temperature acclimation; and (b) low‐temperature acclimation

**Figure 4:**
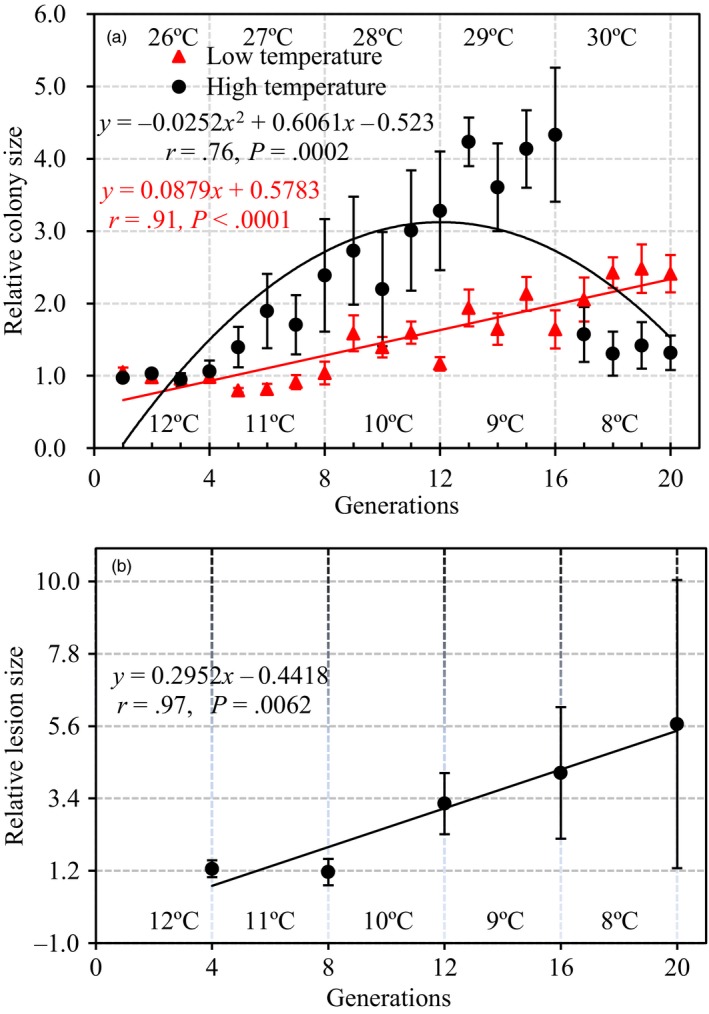
Temporal changes in mean relative colony size, aggressiveness, and their associated 95% confidence intervals of acclimated to unacclimated *Phytophthora infestans* isolates growing in the low‐ and high‐temperature acclimation treatments. The aggressiveness of the isolates was assessed at the end of each four generations, and the mean relative colony sizes and mean aggressiveness were estimated over all 21 isolates collected from the seven locations. Acclimations were carried out in two directions under continuing increase or decrease of temperatures over 20 generations (10 days per generation). At the high‐temperature acclimation, pathogen was continually trained under a steady increase of experimental temperatures at 1°C interval starting from 26°C and ending at 30°C (total five temperature regimes). In each temperature regime, the pathogen was acclimated for four generations. Similarly, at the low‐temperature acclimation, pathogen was continually trained under a steady decrease of experimental temperatures at 1°C interval starting from 12°C. The acclimation was also executed under five temperature regimes (ending at 8°C) each with four generations: (a) relative colony size under high‐ and low‐temperature acclimation; and (b) relative lesion size under low‐temperature acclimation

**Figure 5:**
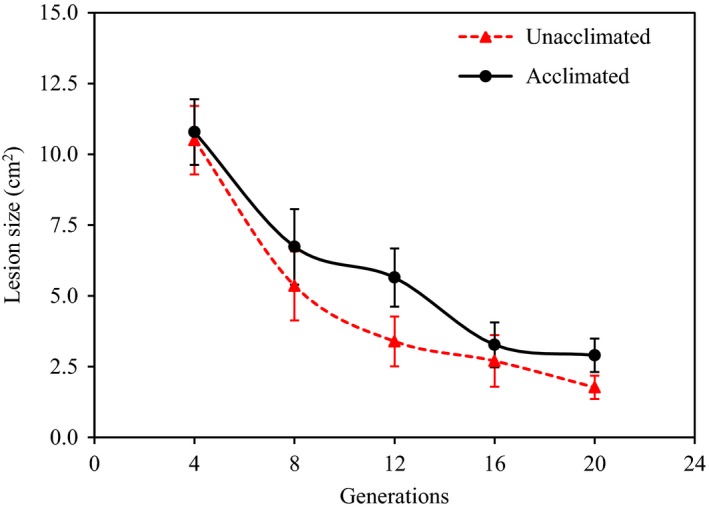
Temporal changes of mean lesion size (aggressiveness) and its associated 95% confidence interval (CI) in the acclimated and unacclimated *Phytophthora infestans* isolates. Aggressiveness of the isolates was assessed at the end of each four generations at a particular temperature regime, and the mean aggressiveness was estimated over all 21 isolates collected from the seven locations

At the temporal scale, no differences in colony size were found between the acclimated and unacclimated parental isolates in the earlier acclimation generations (Figure 3). At high and increasing temperature conditions, thermal adaptation, indicated by an increasing fitness in the acclimated isolates compared to unacclimated isolates, was observed starting from 27°C at the 5th generation of acclimation, speeding up gradually until 29°C at the 16th generation of acclimation and ending sharply after the experimental temperature increased to 30°C (Figure 3a, Figure 4a). At low‐ and decreasing‐temperature conditions, adaptation was observed starting from either the 9th generation at 10°C (colony size)—colony size of unacclimated parental isolates sharply declined at this time—or the 8th generation at 11°C (aggressiveness; Figure 5), and the fitness difference between acclimated and unacclimated parental isolates slowly and waveringly increased as the experimental temperature reduced further (Figures 3b and 4b). In both low‐ and high‐temperature acclimations, more among‐generation fluctuation in colony size was found in acclimated isolates than the unacclimated parental isolates (Figure 3a,b). The pattern of temporal changes in fitness was consistent over the isolates originating from different locations (Figure S1).

**Figure 6:**
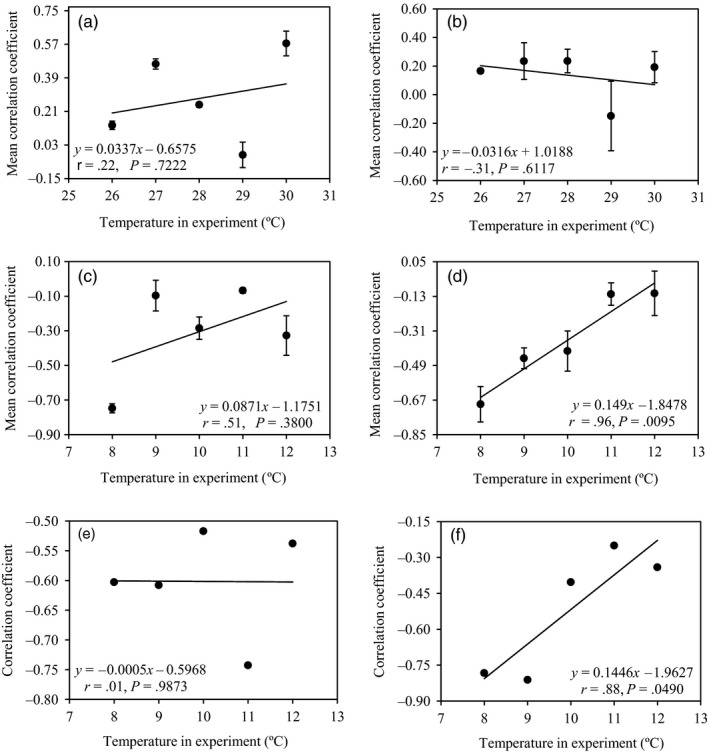
The response of the association between pathogen colony size or lesion size and the annual mean temperature at collection sites to the changing experimental temperatures in acclimated and unacclimated isolates: (a) without high‐temperature acclimation for mycelial growth; (b) with high‐temperature acclimation for mycelial growth; (c) without low‐temperature acclimation for mycelial growth; (d) with low‐temperature acclimation for mycelial growth; (e) without low‐temperature acclimation for aggressiveness; and (f) with low‐temperature acclimation for aggressiveness

**Figure 7:**
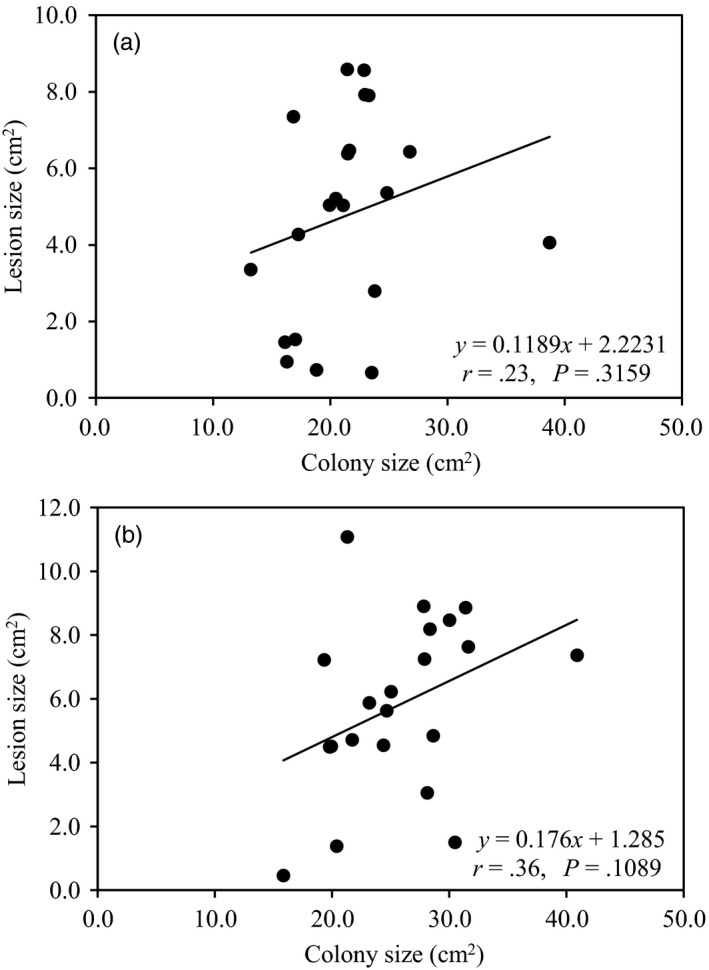
The impact of low‐temperature acclimation on the association between colony size and aggressiveness of 21 *Phytophthora infestans* isolates collected from seven potato field geographic sites in China: (a) unacclimated; and (b) acclimated

Acclimation also changed the pattern of association between performance (colony size and aggressiveness) and annual mean temperature in the collection sites of the pathogen. No association (*r* = .22, *p* = .7222; *r* = −.31, *p* = .6117) between colony size and annual mean temperature at the pathogen collection sites was found in the high‐temperature acclimation (Figure 6a,b). In the low‐ and decreasing‐temperature conditions, the significance of negative association (*r* = .96, *p* = .0095; *r* = .88, *p* = .0490) between performance and annual mean temperature at the pathogen collection sites was strongly affected by experimental temperatures in the acclimated isolates as indicated by a correlation analysis (Figure 6d,f), while no such impact was founded in the unacclimated parental isolates (*r* = .51, *p* = .3800, Figure 6c; and *r* = .01, *p* = .9873, Figure 6e). Similarly, acclimation also affected the association between biological and ecological traits of the pathogen. Higher positive correlation was found between aggressiveness and colony size in the acclimated pathogen compared to the correlation between the two parameters in the unacclimated parental isolates (Figure 7).

## 4 DISCUSSION

Gradual rising global temperature is likely to be one of the major environmental factors significantly affecting future epidemics of infectious diseases such as potato late blight caused by *P. infestans* (Mariette et al., 2016; Sparks et al., 2014; Yang et al., 2016). As a consequence, knowledge of the capacity for thermal adaptation in pathogens is critical to predicting future disease severity and distribution and developing corresponding mitigation strategies (Juroszek & von Tiedemann, 2011). In this study, 21 *P. infestans* genotypes originating from different thermal environments in China were acclimated for 200 days under thermal stresses by a stepwise increasing or decreasing of experimental temperatures. The evolutionary responses of the isolates to these thermal stresses were evaluated by comparing their fitness component (i.e., intrinsic growth rate measured by colony size on petri dishes and aggressiveness measured by lesion size on detached potato leaves) to the parental isolates. Our result show that acclimated *P. infestans* had higher fitness than unacclimated isolates although the extent of this improvement significantly depended on the thermal environments from which the isolates originated (Table 1). Both the intrinsic growth rate and aggressiveness of the isolates increased ~20% in the 200 days of the acclimation experiment (Table 2), resulting in an average of 4% improvement in each step of temperature change.

Both genetic and physiological adaptation can improve the fitness of living organisms surviving in stressing environments (Draghi & Whitlock, 2012; Narum, Campbell, Meyer, Miller, & Hardy, 2013; Somero, 2010). The two events usually occur simultaneously during acclimating processes and are difficult to be fully disentangled. In many experiments involving thermal acclimation, increases in fitness to temperature stresses were thought to be caused primarily by physiological adaptation or phenotypic plasticity (Fierst, 2011; Folguera, Bastías, & Bozinovic, 2009). However, in the current study we have two lines of evidence to support the hypothesis that genetic adaptation to thermal stresses occurred in the pathogen isolates during the 200 days of acclimation.

Empirical studies have shown that physiological adaptation during acclimation usually leads to environmental specialists (Bacigalupe et al., 2018; Levins, 1969). Species perform better in the specific environmental niche to which they were acclimated as compared to their ancestors, but their fitness is not changed or even reduced in other environmental niches to which they were not acclimated. On the other hand, genetic adaptation usually selects for environmental generalists due to heritable change in mean phenotypic value (Chevin, Lande, & Mace, 2010; Walters & Berger, 2019). The adapted species not only demonstrate a higher fitness in the specific environmental niches to which they were acclimated, as relative to their ancestors, but also generally perform better in a wide range of other environmental niches to which they were not acclimated (Rundle & Nosil, 2005). In our experiment, we found that acclimated isolates demonstrated higher fitness at all temperature regimes after five (high temperature) or nine (low temperature) generations of acclimation (Figures 3, 4, 5). In this case, the lag of adaptation time in the lower temperature regimes may reflect its longer generation time relative to the higher temperature regimes. However, it has also been documented that genetic adaptation could lead to thermal specifics (Donelson et al., 2012; Seebacher, Ducret, Little, & Adriaenssens, 2015).

The fitness of the acclimated isolates fluctuated dramatically among generations of the same temperature treatments as compared to the unacclimated ancestry (Figure 3). This pattern of variation is inconsistent with the expectation of a steady increase in fitness caused by physiological adaptation to an acclimating environment (Leroi, Bennett, & Lenski, 1994), but can be generated by genetic adaptation caused by changes of genomic architecture in the acclimated isolates. It was previously demonstrated that genetic adaptation to thermal conditions can reverse or reinforce the effect of physiological adaptation (Ho & Zhang, 2018), leading to the oscillating fitness in the acclimated *P. infestans* isolates among generations of the same temperature treatments. Large genome size and high mutability of the pathogen may contribute to genetic adaptation occurring during this short period of thermal acclimation (Haas et al., 2009; Vetukuri et al., 2012). The pattern of thermal adaptation in the pathogen differed markedly between the high‐ and low‐temperature acclimations (Figure 4). In the low‐temperature acclimation, the fitness of *P. infestans* isolates increased at a later stage and at a slower rate compared to the high‐temperature acclimation. Furthermore, in the low‐temperature acclimation, the fitness of the pathogen isolates was continuingly improved over the entire experiment, while in the high‐temperature acclimation, experimental pathogen isolates suddenly stopped growing when the experimental temperature reached 30°C (Figure 4). The growth collapse point is close to the maximum growth temperature (31.5°C) of the pathogen we previously estimated for these locations (Yang et al., 2016) and suggests a niche boundary may exist in the evolutionary adaptation of *P. infestans* to high‐temperature stresses. Genetic adaptation to extreme stresses requires a range of heritable variation that is broad enough to provide a sustainable growth of living organisms (Huntley, 2007). Lack of time to accumulate further genetic variation may prevent the survival of the pathogen beyond this upper thermal boundary. Unfortunately, we did not continue the experiment after the collapse point and it is not clear whether the pathogen's fitness will recover if acclimation time periods at this temperature regime were extended. At the other end of the thermal scale, the estimated minimum growth temperature for the pathogen isolates included in the acclimation experiments was 5.8°C (Yang et al., 2016). Inevitably there must be a temperature point below which no growth occurs. However, it is not clear at what temperature this might be or whether such a thermal boundary is approached gradually or abruptly although metabolisms usually reduce less steeply when temperature approaches to lower limit than upper limit, leading to skewed thermal curves (Molnár, Sckrabulis, Altman, & Raffel, 2017). Further experiments with a low‐temperature and/or prolonged acclimation to stresses of boundary thermal conditions are required to elucidate these issues.

Thermal adaptation to the low‐temperature acclimation alters the interaction among life‐history and ecological traits in the pathogen as well as the association of these traits with past selection experienced by the pathogen (Figures 4 and 6). For example, low‐temperature acclimation enforces a countergradient adaptation (Yang et al., 2016) of the pathogen to past selection imposed by local temperature regimes (Figure 6c–f) and strengthens the positive association between pathogen's intrinsic growth rate in vitro and aggressiveness (Figure 7). The results suggest that low‐temperature‐acclimated *P. infestans* isolates, and potentially many other pathogens as well, have an enhanced ability to colonize and reproduce on its potato host although a comparative analysis cannot be performed for the high‐temperature acclimation experiment due to the failure to generate aggressiveness data under those temperature regimes.

In conclusion, our study demonstrates that plant pathogen has the ability to rapidly adapt to changing temperature although the underling genetic or physiological mechanisms cannot be disentangled in the current results. In the future study, comparative analyses between acclimated and unacclimated isolates at genetic or genomic level are required to determine relative contribution of genetic and physiological mechanisms to the observed adaptation. A significant feature of the high‐temperature acclimation in the current study was the identification of an apparent thermal mortality threshold for *P. infestans* around 30°C. More importantly though, was the observation that isolates from all collection locations (covering continental, temperate, and subtropical environments) showed significant ability to respond to thermal stresses. While these stresses exceed those currently experienced at the collection sites, they clearly demonstrate *P. infestans* ability to cope with some thermal stresses it is likely to experience in the foreseeable future. Whether these evolutionary trends of pathogen adaptation can be translated into changes in disease severity and distribution under future climatic conditions depends on the relative ability and speed of thermal adaption in the associated hosts (Altizer, Ostfeld, Johnson, Kutz, & Harvell, 2013; Kawecki & Ebert, 2004; Zhan, Thrall, & Burdon, 2014). Therefore, it may not be realistic to robustly predict the future epidemiological landscapes of potato late blight and other main crop diseases without empirical knowledge of thermal adaptation patterns in both plant hosts and pathogens drawn from parallel experiments. However, it is generally believed that pathogens can adapt to temperature shifts more quickly than hosts owing to their faster tissue‐specific metabolisms (Gillooly, Brown, West, Savage, & Charnov, 2001), short generation time (Chen & Stillman, 2012), and larger population sizes (Kingsolver & Huey, 2008; Zhan, Thrall, Papaix, Xie, & Burdon, 2015), and in the case of *P. infestans*, in vitro measurements of aggressiveness in a laboratory conditions are generally correlated well with in vivo data from field trials (unpublished data). These factors enhance the risk of more severe epidemics in the future, and adequate action is required to minimize their impact on food security. Due to the massive work required, we only included three isolates from each of the seven locations in the study. However, the finding of consistent patterns in evolutionary responses among isolates from different locations (Figure S1) suggests that small sample size may not affect our conclusions largely.

## CONFLICT OF INTEREST

None declared.

## Data Availability

Data are available from the Dryad Digital Repository: https://doi.org/10.5061/dryad.z08kprr8h (Wu, Wang, Yahuza, et al., 2019).
