# Supplementary figures and images for: Rapid adaptation of the Irish potato famine pathogen Phytophthora infestans to changing temperature
Source: Evol Appl. 2019 Dec 3;13(4):768–80. doi: 10.1111/eva.12899 (PMC7086108; doi:10.1111/eva.12899)

Supplementary figure S1

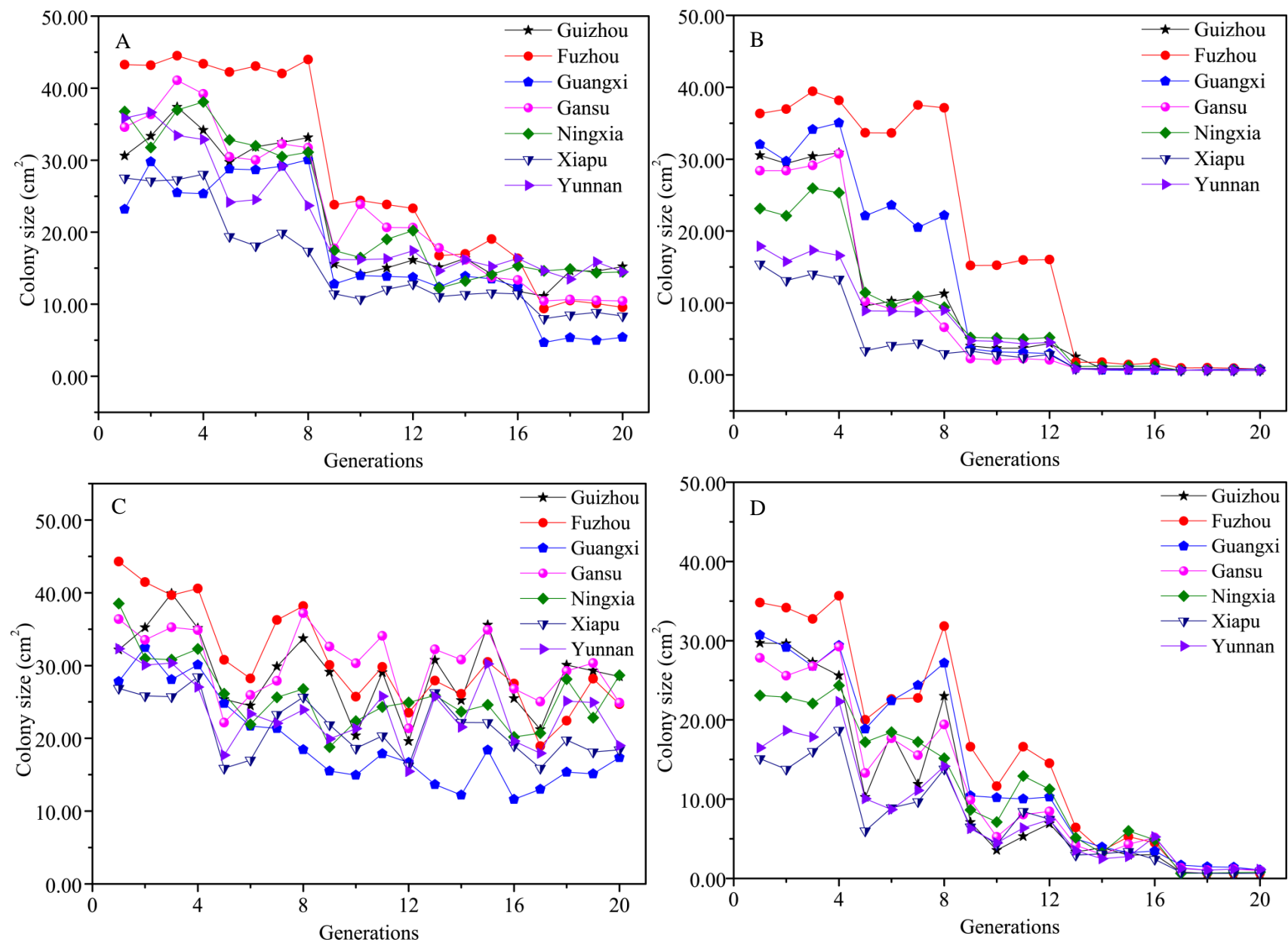

Supplement: Supplementary file 1 [file EVA-13-768-s001.pdf]
