# Supplementary material for: Rapid adaptation of the Irish potato famine pathogen Phytophthora infestans to changing temperature
Source: Evol Appl. 2019 Dec 3;13(4):768–80. doi: 10.1111/eva.12899 (PMC7086108; doi:10.1111/eva.12899)
Supplement: Supplementary file 2 [file EVA-13-768-s002.docx]

Supplementary Figure S1 Temporal changes of mean colony size in the acclimated and unacclimated Phytophthora infestans isolates originating from different locations. (A) Low temperature without acclimation; (B) high temperature without acclimation; (C) low-temperature acclimation; and (D) high-temperature acclimation.
